# Retraction: OsPIE1, the Rice Ortholog of Arabidopsis PHOTOPERIOD-INDEPENDENT EARLY FLOWERING1, Is Essential for Embryo Development

**DOI:** 10.1371/annotation/34c1603f-14a5-4b47-871c-324de18f158a

**Published:** 2011-02-09

**Authors:** Yonghan Xu, Minjuan Deng, Jianfei Peng, Zhanghua Hu, Lieming Bao, Junming Wang, Zhi-Liang Zheng

It has been brought to the attention of the PLoS ONE Editors that Figure 5 in this article was copied from figures originally reported in the following paper published by Hong et al.

Hong SK, Kitano H, Satoh H, Nagato Y. How is embryo size genetically regulated in rice? Development. 1996 Jul;122(7):2051-8.

The first author, Dr Yonghan Xu, has indicated that the copying was done by a member of his group at Zhejiang Academy of Agricultural Science without the knowledge of the other authors.

PLoS ONE and the authors therefore retract this article due to the concerns raised over the results reported in the article.

The authors would like to apologise to readers, the authors of the original article, and to the editors and publisher of Development.

No competing interests declared. 

